# Diagnosing Organic Causes of Schizophrenia Spectrum Disorders: Findings from a One-Year Cohort of the Freiburg Diagnostic Protocol in Psychosis (FDPP)

**DOI:** 10.3390/diagnostics10090691

**Published:** 2020-09-14

**Authors:** Dominique Endres, Miriam Matysik, Bernd Feige, Nils Venhoff, Tina Schweizer, Maike Michel, Sophie Meixensberger, Kimon Runge, Simon J. Maier, Kathrin Nickel, Karl Bechter, Horst Urbach, Katharina Domschke, Ludger Tebartz van Elst

**Affiliations:** 1 Section for Experimental Neuropsychiatry, Department of Psychiatry and Psychotherapy, Medical Center—University of Freiburg, Faculty of Medicine, University of Freiburg, 79104 Freiburg, Germany; dominique.endres@uniklinik-freiburg.de (D.E.); miriam.matysik@uniklinik-freiburg.de (M.M.); bernd.feige@uniklinik-freiburg.de (B.F.); tina.schweizer@uniklinik-freiburg.de (T.S.); sophie.marie.meixensberger@uniklinik-freiburg.de (S.M.); kimon.runge@uniklinik-freiburg.de (K.R.); simon.maier@uniklinik-freiburg.de (S.J.M.); kathrin.nickel@uniklinik-freiburg.de (K.N.); 2 Department of Psychiatry and Psychotherapy, Medical Center—University of Freiburg, Faculty of Medicine, University of Freiburg, 79104 Freiburg, Germany; maike.michel@uniklinik-freiburg.de (M.M.); katharina.domschke@uniklinik-freiburg.de (K.D.); 3 Department of Rheumatology and Clinical Immunology, Medical Center—University of Freiburg, Faculty of Medicine, University of Freiburg, 79106 Freiburg, Germany; nils.venhoff@uniklinik-freiburg.de; 4 Department for Psychiatry and Psychotherapy II, Ulm University, Bezirkskrankenhaus Günzburg, 89312 Günzburg, Germany; karl.bechter@bkh-guenzburg.de; 5 Department of Neuroradiology, Medical Center—University of Freiburg, Faculty of Medicine, University of Freiburg, 79106 Freiburg, Germany; horst.urbach@uniklinik-freiburg.de; 6 Center for Basics in Neuromodulation, Faculty of Medicine, University of Freiburg, 79106 Freiburg, Germany

**Keywords:** schizophrenia, psychosis, autoimmune psychosis, screening, antibody, cerebrospinal fluid, EEG, MRI

## Abstract

**Introduction:** Secondary schizophrenia spectrum disorders (SSDs) have clearly identifiable causes. The Department for Psychiatry and Psychotherapy at the University Hospital Freiburg has continued to expand its screening practices to clarify the organic causes of SSDs. This retrospective analysis was carried out to analyze whether a comprehensive organic diagnostic procedure could be informative in patients with SSDs. **Methods and Participants:** The “Freiburg Diagnostic Protocol in Psychosis” (FDPP) included basic laboratory analyses (e.g., thyroid hormones), metabolic markers, pathogens, vitamin status, different serological autoantibodies, rheumatic/immunological markers (e.g., complement factors), cerebrospinal fluid (CSF) basic and antineuronal antibody analyses, as well as cranial magnetic resonance imaging (cMRI) and electroencephalography (EEG). The findings of 76 consecutive patients with SSDs (55 with paranoid–hallucinatory; 14 with schizoaffective; 4 with hebephrenic; and 1 each with catatonic, acute polymorphic psychotic, and substance-induced psychotic syndromes) were analyzed. **Results:** Overall, vitamin and trace element deficiency was identified in 92%. Complement factor analyses detected reduced C3 levels in 11%. Immunological laboratory alterations were detected in 76%. CSF analysis revealed general alterations in 54% of the patients, mostly with signs of blood–brain barrier dysfunction. cMRI analyses showed chronic inflammatory lesions in 4%. Combination of EEG, cMRI, and CSF revealed alterations in 76% of the patients. In three patients, autoimmune psychosis was suspected (4%). **Discussion:** On the basis of these findings, we conclude that a comprehensive diagnostic procedure according to the FDPP in patients with SSD is worthwhile, considering the detection of secondary, organic forms of SSDs, as well as alterations in “modulating factors” of the disease course, such as vitamin deficiency. Larger studies using comprehensive diagnostic protocols are warranted to further validate this approach.

## 1. Introduction

Schizophrenia spectrum disorders (SSDs) are characterized by delusions, hallucinations, disorganized speech, affective flattening, and cognitive deficits that are linked to significant disability and psychosocial impairment. They also are associated with psychosocial stigma and discrimination [1,2,3]. In primary, idiopathic forms of SSDs, examination findings are typically inconspicuous, and a genetic liability is assumed [4,5]. However, secondary schizophrenia spectrum disorders (SSSDs) have clearly identifiable organic causes and can occur in the context of central autoimmune (e.g., autoimmune encephalitis, autoimmune psychosis) or systemic autoimmune (e.g., systemic lupus erythematosus), infectious (e.g., neuroborreliosis, neurosyphilis), endocrine (e.g., hyperthyroidism, hyperparathyroidism), metabolic (e.g., Wilson’s disease, metachromatic leukodystrophy), epileptic (e.g., temporal lobe epilepsy), cerebrovascular (e.g., strategic infarcts), genetic (e.g., velocardiofacial syndrome, Niemann—Pick disease type C), tumor-related (e.g., gliomas), neurodegenerative (e.g., Huntington’s disease), substance-related (e.g., cannabis, phencyclidine), or dietary-related (e.g., vitamin B12 deficiency) diseases [6]. In addition to clinical evaluation, a number of other examinations are available to investigate secondary causes. The most frequently used are blood analyses (for testing antineuronal antibody or hormone levels, karyotyping, etc.), urine examinations (drug screening, markers for porphyria, etc.), structural imaging (cranial magnetic resonance imaging (cMRI), etc.), electroencephalography (EEG; routine/sleep EEG, etc.), and cerebrospinal fluid (CSF) analyses (basic diagnostics, antibody/pathogen detection, etc.). In the case of specific suspicion, further examinations, such as neuronuclear methods ([18^F^]fluorodeoxyglucose positron emission tomography (FDG-PET), etc.); electrophysiological examinations (evoked potentials, etc.); or, more rarely, invasive methods (brain biopsies, etc.) can be performed [7]. Country-specific guidelines provide recommendations on which diagnostic regime should be used to exclude secondary causes. The German S3 guideline recommends obligatory complete physical and neurological examinations, several blood tests (e.g., C-reactive protein (CRP) or thyroid parameters), drug screening in urine, and cMRI. CSF and EEG analyses, as well as further blood examinations (e.g., for brain-reactive autoantibodies), are suggested in cases of specific suspicion based on clinical findings and baseline examinations [8]. In recent years, increased emphasis has been put on the detection of autoimmune encephalitis and autoimmune psychoses, in particular, as differential diagnosis for SSDs [7,9,10,11]. As affected patients frequently respond to treatment with anti-inflammatory drugs, an optimal and early diagnosis is essential [7]. Following our experiences that secondary, organic psychoses cannot always be reliably differentiated from primary, idiopathic variants on the basis of the baseline examinations mentioned above [5,12,13,14,15], the Department of Psychiatry and Psychotherapy at the University Hospital Freiburg, Germany, has been continuously expanding and refining its diagnostic protocol of screening for organic causes of psychotic disorders (“Freiburg Diagnostic Protocol of Psychoses” (FDPP)). Since 2018, patients on the specialized ward of the Department of Psychiatry and Psychotherapy, University of Freiburg, have been offered broad laboratory screening, as well as cMRI, EEG, and CSF analysis.

### Rationale of the Current Study

The FDPP extends far beyond the current German S3 and other guidelines’ recommendations. The present retrospective analysis was performed to analyze whether such a broad organic screening is acceptable to patients and whether it is informative in all patients with SSDs. Moreover, we wanted to test whether or not the respective findings from such an extensive diagnostic procedure might justify the efforts or alternatively support the foreseeable criticism that this diagnostic protocol is exaggerated. The main hypothesis of our retrospective workup was that, first, autoimmune psychoses and other organic forms can be detected using the FDPP, and second, a relevant number of alterations can be found in disease-modulating factors, such as neurovitamins.

## 2. Materials and Methods

In 2018, 76 patients over 18 years who suffered from SSDs were examined on the specialized ward for psychoses, autism spectrum disorders, and organic psychiatric disorders. Data of these patients were retrospectively analyzed in the current project, which was approved by the local ethics committee (Faculty of Medicine, University of Freiburg, ethical vote no. 396/18).

### 2.1. Patient Cohort

A search was made for patients with the following diagnoses (classified by experienced senior psychiatrists according to the criteria of the International Classification of Diseases, 10th revision), who were treated on our special ward in 2018: organic delusional (schizophrenia-like) disorder (F06.2), substance-induced psychotic disorder (F1x.5), schizophrenia (F20.x), persistent delusional disorders (F22.x), acute and transient psychotic disorders (F23.x), and schizoaffective disorders (F25.x; https://www.who.int/classifications/icd/en/GRNBOOK.pdf?ua=1). For further analysis, the patients were grouped according to their predominating clinical syndrome. The categorization was made in terms of paranoid-hallucinatory, schizoaffective, hebephrenic, catatonic, acute polymorphic psychotic, substance-induced psychosis, and delusional syndromes. We also distinguished between acute and relapsing/chronic forms of the disease. The 76 patients included in this study suffered from the following syndromes: 55 (72%) from paranoid–hallucinatory, 14 (18%) from schizoaffective, 4 (5%) from hebephrenic, 1 (1%) from catatonic, 1 (1%) from acute polymorphic psychotic, and 1 (1%) from substance-induced psychosis syndromes; no patient suffered from a delusional syndrome (0%). A total of 15 patients were investigated during the first episode (20%), and 61 cases had a chronic or recurrent disorder (80%).

### 2.2. Blood and Cerebrospinal Fluid Analyses

The investigations carried out are summarized in Table 1. Blood examinations were performed according to established methods and as part of clinical routine. Most analyses were performed in the Central Laboratory of the University Hospital Freiburg. Blood tests were performed in all 76 patients, but not all parameters were carried out in each patient during clinical work-up. CSF analyses were performed in the CSF laboratory of the Department of Neurology of the University Hospital Freiburg in accordance with international recommendations [16]. Tests for antibodies against neuronal cell surface antigens and anti-AQP4/MOG antibodies were performed using fixed cell biochip assays from Euroimmun^®^. The antineuronal antibodies against intracellular antigens were investigated using Ravo line assays^®^. Anti-glutamate decarboxylase (GAD) 65 antibodies were also tested using radioimmunoassay (RIA). Methods for rheumatic testing and CSF basic analyses are described in earlier papers from our working group [14,17]. CSF analyses were performed in 48 of the 76 patients (63%) in 2018.

### 2.3. Electroencephalography

EEGs were performed in all 76 patients (100%), while MRI investigations of the brain were carried out in 74 patients (97%) (Table 1). EEG findings were evaluated by the physician responsible for the respective patient and alterations were retrospectively coded as one of the following: continuous generalized slow activity, continuous regional slow activity, intermittent rhythmic generalized delta/theta activity (IRDAs/IRTAs), intermittent regional slow activity, or epileptic activity. The EEGs were also evaluated automatically and examined for IRDAs/IRTAs. The methodology has been described in detail in earlier papers [18,19].

### 2.4. Cerebral Magnetic Resonance Imaging

We routinely acquired T1-weighted (axial 5 mm thick fast spin echo slices on a 1.5 Tesla, magnetization prepared rapid gradient echo (MPRAGE) sequences with isotropic 1 mm^3^ voxels on a 3 Tesla scanner), diffusion-weighted imaging (DWI) (axial 5 mm thick slices), and fluid-attenuated inversion recovery (FLAIR) sequences (coronal 3 mm thick fast spin echo slices on a 1.5 Tesla, 3D SPACE sequence with isotropic 1 mm^3^ voxels on a 3 Tesla scanner). The MPRAGE sequence is routinely used for voxel-based analysis and thus helps to detect regional atrophy. The FLAIR sequence is needed to detect increased signal intensity, particularly of the mesiotemporal structures that are often also swollen in limbic encephalitis. DWI may detect acute or subacute (strategic) infarcts and is the most sensitive sequence for the diagnosis of Creutzfeldt–Jakob disease. All cMRIs were evaluated by experienced neuroradiological specialists. The cMRI alterations were divided into five categories: white matter (WM) changes (with any small, non-specific WM lesion rated as an alteration), (chronic) inflammatory lesions, atrophy (local or generalized), pineal cysts, and others.

### 2.5. Statistics

The data analysis was performed using SPSS, version 24 (IBM, New York, NY, USA). The findings were mainly reported descriptively. The rate of pathological values was based on established reference values. In addition, a Pearson correlation between CSF basic parameters (white blood cell (WBC) count, protein concentration, albumin quotient, and immunoglobulin (Ig) G index) and IRDA/IRTA rates was performed. A *p*-value of <0.05 was assumed to be significant. Due to the exploratory approach, no correction for multiple testing was performed.

## 3. Results

### 3.1. Sociodemographic Data

Patients with SSDs were on average 37 years old and predominantly female (58%). At the time of the initial laboratory examination, 83% of the patients with SSDs were pharmacologically treated. Table 2 gives an overview of the sociodemographic data and medication.

### 3.2. Laboratory Findings

#### 3.2.1. Basic Laboratory Findings

The following alterations in basic laboratory analyses were detected in the patients with SSDs (Table 3):

**Blood count, coagulation, electrolytes:** Of all patients, 14% showed increased hemoglobin value. The partial thromboplastin time (PTT) values were increased in 5% and decreased in 9% of the patients. Calcium levels were elevated in 12% of the patients, whereas magnesium concentrations were within the normal range in all patients.

**Metabolic markers:** The alpha-galactosidase levels were reduced in 11% of the patients. The creatine kinase (CK) value was increased in 15%. All except one patient with elevated liver values or elevated alkaline phosphatase received psychopharmacological treatment.

**Thyroid hormones:** The thyroid-stimulating hormone (TSH) values were increased in 5%, and the free thyroxine (fT4) value was changed in 9% of the patients. The free triiodothyronine (fT3) was in the normal range in all patients. Overall, changes in thyroid hormones were observed in 12% of the patients.

#### 3.2.2. Vitamins/Trace Elements

Vitamin D levels were below 20 ng/mL in 46%, and below 30 ng/mL in 84%; the folic acid levels were lowered in 32%; and the vitamin B12 value was decreased in 1% of the patients. The selenium values were reduced in 80%. Overall alterations in vitamins and trace elements were detected in 92% of the patients (Table 4).

#### 3.2.3. Pathogen Diagnostics

In the Western blot, no patient showed evidence of acute Lyme disease, although 7% displayed evidence of a previous Lyme disease infection. The lues serology was positive in one patient (1%), however, the activity parameters and the confirmatory test were unremarkable in this patient (Table 5). Thus, there was no indication of an acute pathogen-related cause of schizophreniform symptomatology.

#### 3.2.4. Immunological Serum Screening

*Rheumatic/immunological markers:* The CRP was elevated in 17% of the patients. Immunoglobulin (Ig)M concentrations were changed in 10%, and the immunofixation was altered in 8%. C3 was decreased in 15%, and C4 was decreased in 1%. C3d was elevated in 12% of the patients. The rheumatoid factor was essentially normal in all patients.

*Brain-associated systemic antibodies:* Anti-thyroglobulin (TG) antibodies were elevated in 4%, and anti-thyroid peroxidase (TPO) antibodies in 12%. One female patient with paranoid-hallucinatory syndrome and anti-TPO antibodies, with EEG slowing, increased albumin quotient, and cMRI atrophy responded well to steroids and was therefore re-diagnosed with Hashimoto encephalopathy (the diagnosis was already made 2017, in 2018 the symptomatology worsened again, which is why the patient was again treated as an inpatient). The anti-GAD65 antibodies measured by RIA were elevated in one patient (1%). The anti-GAD65 antibody-positive patient suffered from catatonic schizophrenia; his EEG and cMRI showed no abnormalities, and CSF analyses only showed increased total protein. The anti-GAD65 antibodies were evaluated as non-specific in this patient.

*Potential antineuronal-rheumatic and other rheumatic serum antibodies:* Antinuclear antibody (ANA) reactivity was found in 15% of the patients. Extractable nuclear antigen (ENA) differentiation was clearly positive in three patients, one of these patients had anti-snRNP/Sm antibodies and the other two had anti-DFS70 antibodies. The patient with anti-snRNP/Sm antibodies was re-diagnosed with mixed connective tissue disease on the basis of the findings of discrete interstitial lung disease, Raynaud’s syndrome, and probable brain involvement, as documented by abnormal EEG and FDG-PET findings. Screening for anti-neutrophil cytoplasmic antibodies (ANCAs) by indirect immunofluorescence (IIF) was positive in 3% of the patients; however, specificity against myeloperoxidase (MPO) and proteinase 3 (PR3) by ELISA testing was not found. The antiphospholipid (ß2-glycoprotein-1) IgG and/or IgM antibodies were slightly elevated in 10%. Non-specific white matter lesions were detected in cMRI in four of seven patients with anti-phospholipid antibodies (57%).

*Antineuronal serum IgG antibodies:* Testing for the established paraneoplastic antibodies against intracellular antineuronal antigens showed clear positive antibodies against anti-Yo in one patient (1%). This patient suffered from paranoid schizophrenia; his EEG showed intermitted generalized slow activity, his cMRI/brain FDG-PET showed no abnormalities, and CSF was not investigated (rejected by the patient). In whole body computer tomography/FDG-PET no tumor was detected. The significance of the anti-Yo antibodies remained unclear in this patient, due in part to the missing of CSF results. Six patients showed weak non-specific positive bands for Yo (three patients), SOX1 (two patients), and Zic4 (one patient; overall 8%). All screenings for antibodies against established antineuronal cell surface antigens and anti-aquaporin 4 (AQP4) were negative in this cohort. The anti-myelin oligodendrocyte glycoprotein (MOG) antibodies were positive in one patient with atypical paranoid–hallucinatory syndrome (2%). The EEG revealed a dysrhythmic alpha rhythm, while the cMRI showed non-specific white matter lesions. His CSF analyses indicated increased total protein. The anti-MOG antibody finding was repeatedly positive (also with a live cell-based assay) and was classified as of possible pathophysiological relevance, which led to the diagnosis of a potential autoimmune psychosis. For details, please see Table 6.

### 3.3. Cerebrospinal Fluid Findings

CSF analyses were performed in 48 of 76 patients, corresponding to a rate of 63%. Two of the patients without CSF results received a lumbar puncture in the course of the disease. Therefore, a total of 66% of the patients agreed to lumbar puncture. The CSF WBC count was not increased in the included sample. One increased WBC count was caused by blood admixture, the corrected value was normal (correction formula: 1 cell/µL of WBC count reduction per 1000 red blood cells/µL). The total protein was elevated in 56% of the patients. The albumin quotients were increased in 21%. The IgG index was increased in 4%, and oligoclonal bands (OCBs) in the CSF were found in one patient (2%). In 45% of the patients, the lactate levels were below the lower reference value. None of the patients displayed antineuronal antibodies against the established antigens in the CSF. Overall, CSF alterations (without reduced lactate) were detected in 54% of the patients (for details, see Table 7).

### 3.4. Instrument-Based Diagnostics

The EEGs indicated alterations in 29% of the patients, most frequently with intermittent generalized slow activity (IRDAs/IRTAs). In the cMRI, alterations were found in 61% of the patients, most frequently with non-specific white matter lesions (in 38%; if each singular lesion was assessed as altered). Pineal cysts were detected in 16% and chronic inflammatory lesions in 4%. For details, please see Table 8.

### 3.5. Overall Alterations

Immunological blood alterations were detected in 76% of the patients. Vitamin and trace element deficiency was detected in 92%. Overall CSF alterations (without decreased lactate) were found in 54% of the patients. The combination of EEG, cMRI, and CSF analysis resulted in alterations in 76% of the patients. Autoimmune psychosis was suspected in three patients (4%; one with Hashimoto encephalopathy, one with anti-MOG antibody-associated psychosis, and one with mixed connective tissue disease with probable brain involvement). Another patient, who was not included in this analysis because he was under 18 years of age in 2018 (exclusion criterion), had an autoimmune psychosis with novel antineuronal antibodies in the tissue test.

### 3.6. Correlation Analyses

CSF WBC count correlated significantly with IRDAs per minute (*r* = 0.336, *p* = 0.019), with IRDAs per minute before hyperventilation (*r* = 0.399, *p* = 0.005) and with IRDAs per minute after hyperventilation (*r* = 0.333, *p* = 0.029). Other than that, there were no significant correlations between CSF markers and IRDA/IRTA rates.

## 4. Discussion

In this paper, we present the Freiburg Diagnostic Protocol in Psychosis (FDPP) and show that it could produce relevant findings in a substantial subgroup of patients with SSDs. Applying FDPP screening, autoimmune psychosis was suspected in 4% and immunological blood alterations were detected in 76% of patients with SSDs. The combination of EEGs, cMRIs, and CSF also resulted in alterations in 76% of the patients. Modulating factors for the course of the disease (neurovitamins and trace elements) revealed levels below the recommended reference values in 92% of all patients. On the basis of these findings, we feel that such an extended screening approach in patients with SSDs could be worthwhile. Anticipating criticisms, as the majority of findings might not be clinically relevant, we want to discuss the significance of these alterations, but also possible limitations, for the detection of SSSDs and specific treatment below.

### 4.1. Blood Analyses

Surprisingly, the basic laboratory findings showed signs of hypercalcemia in 12% of the patients. These findings could indicate endocrinological disorders (e.g., primary hyperparathyroidism, hyperthyroidism, or adrenal cortex hypofunction), malignant tumors (e.g., plasmocytoma, paraneoplastic processes), or sarcoidosis [20], but also can occur due to lack of exercise. There were reduced levels of α-galactosidase in 11%; this could indicate Fabry’s disease, an X-linked recessive lipid storage disease characterized by α-galactosidase deficiency, which could be confirmed with a mutation analysis. The association between Fabry’s disease and depression is known, and an association with psychoses has been discussed [21,22]. Given these findings, the question arises whether there might be minor variants of metabolic diseases such as Fabry’s disease that could be of causal relevance in the development of mental disorders. This hypothesis cannot be answered presently since there is still too little research done in this area. In our patients and on the basis of present knowledge, we made the clinical judgment that they did not suffer from full blown Fabry’s disease because the currently accepted critical symptoms were not present (strokes, polyneuropathies, angiokeratomas, renal insufficiency; [23]); however, we recommended that patients undergo genetic testing. Alternatively, alpha-galactosidase deficiency could have been induced by antipsychotics. The frequently increased transaminases (GOT in 13%, GPT in 26%), γ-GT (in 17%), and alkaline phosphatase (in 16%) were most likely due to psychotropic drugs.

Vitamin and trace element analyses revealed deficiencies in 92% of the patients. Suboptimal vitamin D levels were detected in 84%, and reduced folic acid concentrations were present in 32%. Vitamin D seems to have both immunomodulatory effects, via its effect on T and B lymphocytes, and neuroprotectant and neurotrophic effects [24]; therefore, substitution therapy is recommended in patients with multiple sclerosis and rheumatic diseases. The significance of vitamin D deficiency for SSDs is currently the subject of intense discussion [25]. In patients with deficiency, we offered substitution of vitamin D. Folic acid is responsible for cell division and cell maintenance [26], and a deficiency can lead to leukoencephalopathy with psychiatric symptoms [27]. In patients with SSDs, a substitution with folic acid might be useful in reducing negative symptoms [28]. In patients with deficiency, we suggested substitution of folic acid. Selenium levels were below the recommended limits in 80% of the patients, which is in line with the findings of a previous study on schizophrenia [29]. Selenium deficiency appears to be associated with an increased risk of autoimmune thyroiditis; selenium can reduce thyroid antibody levels and may exert its immunomodulatory effects by reducing pro-inflammatory cytokines [30]. In patients with deficiency, we offered substitution of selenium. The vitamin or selenium deficiency states could not only be causally involved in the development of the schizophreniform symptoms, but could also be secondary (e.g., a vitamin D deficiency could be caused by avoiding sunlight through social withdrawal).

The most common anti-thyroid antibodies were directed against TPO, which were elevated in 12% of the patients. These antibodies may indicate autoimmune thyroiditis, but, after exclusion of antineuronal autoantibodies, they might, in rare cases, also be associated with Hashimoto encephalopathy in patients with unclear cases of autoimmune encephalitis [9,10], as was probably the case with one patient of the present cohort. Low levels of positive anti-GAD56 antibodies are found in diabetes mellitus, however, in our cohort, one patient had increased concentrations of anti-GAD65 antibodies in RIA without diabetes mellitus. Increased levels of anti-GAD65 antibodies are found more frequently in patients with psychosis than in control groups [31] and can be associated with limbic encephalitis in a subgroup of the patients with high titers; however, their pathophysiological role is not yet clear [32]. The rheumatic screening showed CRP increases in 17% of the patients. Slightly elevated CRP levels have been described on a meta-analytical level, and elevated CRP values support the idea of systemic low-grade inflammation in a subgroup of the patients with SSDs [33]. Complement factor analysis showed frequently decreased levels of C3 (15%). Lowered C3 concentrations are classically found in patients with immune complex diseases, such as systematic lupus erythematosus [34], which in turn can mimic psychoses [35]. As one molecular genetic investigation in schizophrenia identified a complement factor C4 gene variant as a potential genetic marker [36], there may be a direct association between schizophrenia and the complement system.

The rheumatoid factor was normal in all patients. This finding is compatible with the results of large studies, which show a negative correlation between SSD and rheumatoid arthritis. The underlying pathophysiological processes are not conclusively clarified [37] but could possibly be caused by aberrant inflammatory cytokine profiles [38]. Anti-phospholipid antibodies were slightly increased in 10% of the patients. The presence of anti-phospholipid antibodies in some patients with schizophrenia is known [39], and SSD may also occur in patients with fully developed anti-phospholipid syndrome [40]. Conspicuous ANAs were found in 15% of the patients. Other studies showed comparable findings (e.g., [41]); these “potentially antineuronal” antibodies may be a sign of connective tissue diseases. Clear evidence of connective tissue diseases was found in extractable nuclear antigen (ENA) testing, with anti-snRNP/Sm specificity in one patient, who was subsequently re-diagnosed with mixed connective tissue disease with suspected brain involvement. In two patients, anti-DFS70 antibodies were found, indicating the absence of ANA-associated rheumatic diseases [42]. In addition to one patient with anti-GAD65 antibodies in the RIA, there were two other cases where testing clearly revealed positive results for antineuronal serum antibodies. One patient with schizophreniform syndrome had anti-Yo antibodies in the serum, while the additional findings revealed no clear signs for brain involvement. Another patient with atypical psychosis, as well as EEG and cMRI alterations, was tested positive for anti-MOG antibodies. In the context of the presently expanding spectrum of neuromyelitis optica (NMO) spectrum disorders [43], we suspected a potential autoimmune psychosis.

### 4.2. CSF Markers

Overall, 66% of patients agreed to a lumbar puncture. The patients were only examined after their written informed consent, and no emergency LPs were performed against the will of the patients. This shows relatively high acceptance of this procedure on our specialized ward and demonstrates that lumbar punctures can be successfully performed in a psychiatric setting. The CSF findings showed evidence of inflammatory changes in 4% (increased IgG indices in two patients, with OCBs in one patient), while a blood–brain barrier dysfunction with increased albumin quotients was observed in 21% (elevated CSF protein levels have even been reported in >50% of patients, but on the basis of current knowledge they are no longer suggested as optimal markers of blood–brain barrier dysfunction, and albumin ratios should be preferred as they better reflect the underlying decreased CSF flow rate; [44,45]). WBC counts correlated with IRDA/IRDA rates in EEGs. While inflammatory change frequency was lower than in previous studies, the frequency of disturbed blood–brain barrier parameters was previously described with similar figures [14,46,47]. The blood–brain dysfunction’s role in the pathophysiology of schizophreniform psychosis is currently under intense discussion (see [48,49]). The established antineuronal IgG antibodies against cell surface antigens were negative in the patients studied here, which aligns with the findings of Oviedo-Salcedo and colleagues (2018), using the same methodology [50]. However, in 2018, no screening using tissue-based assays using indirect immunofluorescence screening on brain sections of rodents were performed on our ward. It was not until the end of 2018 that we started to do this and, in the meantime, detected some pathological findings that led to the diagnosis of possible autoimmune psychosis (e.g., [12,13]) in patients in whom the overall clinical judgment would sometimes not have led to this diagnosis without this finding. Novel autoantibodies against still-unknown antigens, which we unfortunately did not investigate here, may play a more important role than established antineuronal antibodies against cell surface antigens in psychiatric diseases.

### 4.3. Instrument-Based Diagnostics

EEG alterations were detected in 29% of the patients, and (mostly non-specific) cMRI alterations in 61%. IRDAs/IRTAs were the most frequently detected alterations using EEG (in 21%). Earlier studies showed similar findings [14,51]. These EEG alterations may be an indication of neuronal network instability (see local area network inhibition hypothesis; [52]). Clinically they might be a finding that supports the consideration of anticonvulsive therapy trials [52]. The most common cMRI alteration was the presence of non-specific white matter changes (in 38%) when evaluating each small lesion. However, only in 4% of patients were chronic inflammatory lesions detected. White matter lesions may be associated with neuroinflammatory processes [53], but non-specific changes can often also be found in healthy controls [54]. Pineal cysts were found in 16% of the patients; these findings were usually interpreted as non-specific. However, the prevalence in our cohort seems to be much higher than in controls. For example, in one imaging study of 1400 healthy control subjects, the prevalence was only 2% [55]. The significance of pineal cyst findings in patients with SSDs is unclear. One case report describing an improvement of psychotic symptoms after resection of a pineal epidermoid cyst in a 23-year-old male patient [56] indicates that such incidental findings might be of clinical relevance at least in some patients. Thus, whether there is a pathophysiological association between pineal cysts and psychosis should be investigated in future studies.

### 4.4. Overall Alterations

Earlier studies have described a clear bidirectional association between autoimmune diseases and SSDs [57,58,59]. In this study, slight immunological alterations were found in the serum of 76% of the patients. The combination of EEG, cMRI, and CSF also showed abnormalities in 76% of the patients. In some cases, this led to the clear diagnosis and treatment of a concomitant disorder (e.g., in a patient with anti-snRNP/Sm antibodies who was diagnosed with mixed connective tissue disease). In other cases, the significance of more singular findings such as only EEG slowing, cMRI white-matter lesions, or CSF abnormalities remained unclear. This common clinical constellation with only one pathological but still non-specific abnormal finding is difficult to interpret for the clinician and clearly warrants more intensive clinical research in this area, whereas frequent deficiencies of vitamins and selenium can be substituted.

### 4.5. Limitations

The results are limited by the uncontrolled, open, and retrospective design. Due to the uncontrolled design, no comparison with a healthy or psychiatric control group was possible, although laboratory and CSF values were compared to established reference values. However, therefore it cannot be clarified whether the current findings are disease-specific results for patients with schizophreniform disorders. Due to the open and retrospective design, many findings were not available for the entire patient sample. The open and university setting and the relatively small sample size also may have led to a distortion (selection bias) of the results. The screening suggested here was based on our own clinical experiences and was adapted over the last few years. For example, the MRZ reaction was also recorded temporarily, but this did not have relevant clinical consequences and was not further investigated in the course of the screening [60]. Further blood markers (e.g., ceruloplasmin, broader pathogen search, genetic testing) or additional investigations (e.g., FDG-PET) could be useful in some patients (see Table 1). With regard to the analysis of antineuronal antibodies against cell surface antigens in serum and CSF, which were completely negative in the presented patient group, more sensitive methods (live cell-based assays and tissue-based assays using indirect immunofluorescence on fixed/unfixed murine brain tissue) could be of higher clinical benefit [12,13,61].

## 5. Conclusions

In this paper, the authors presented the concept of the FDPP screening, a comprehensive, multimodal diagnostic screening approach that includes broad laboratory testing in blood and CSF, as well as cMRI and EEG investigations, designed to exclude secondary, organic causes in patients with SSDs. Lumbar punctures were performed in well over half the cases. Thus, our data show that implementation of lumbar punctures in a psychiatric setting is possible. In contrast to the recommendations of most international guidelines, FDPP screening includes comprehensive diagnostic screening measures that generally are only sought in cases with clear clinical evidence of such secondary disorders. On the basis of our clinical experience that secondary variants of psychotic disorders may present like primary, idiopathic SSDs [12,13,15], we developed the FDPP approach at our center in recent years. The study shows that a broad organic assessment in patients with psychosis could be helpful in detecting secondary, organic psychoses. Our study indicates that with the FDPP approach, autoimmune psychosis can be found in about 4% of patients and that alterations in modulating factors such as neurovitamins and trace element alterations are found in about 92%. The interpretation of other non-specific findings (e.g., isolated weakly elevated anti-phospholipid antibodies, isolated IRDAs/IRTAs in EEG) requires further research. Larger and controlled studies in the future could help refine this multimodal screening approach in an effort to identify and differentially treat patients with primary idiopathic presentation of what then turns out to be SSSD. On the basis of our clinical experience, most patients and relatives welcome this approach, even in the case of negative or unclear findings. With respect to this approach’s costs, we feel that this is not relevant, given the severity of the diseases in question and the far-reaching psychosocial and occupational consequences that a faulty diagnosis of primary, idiopathic SSD might hold.

## Figures and Tables

**Table 1:** Screening approach in patients with schizophrenia spectrum disorders.

		Freiburg FDPP Screening	Additional Analyses in Selected Cases
**Extended basic laboratory analyses**	**Blood count**	-- Differential blood count	-- Acanthocytes ^1^
	**Coagulation**	-- INR/Quick, PTT	-- Lupus anticoagulant ^2^
	**Electrolytes**	-- Sodium, potassium, calcium, magnesium	
	**Metabolic markers**	-- Creatinine, CK, GOT, GPT, AP, γ-GT, lipase -- HbA1C, total triglycerides, total cholesterol, homogeneous LDL cholesterol, homogeneous HDL cholesterol Alpha-galactosidase ^3^	-- Parathyroid hormone ^4^, phosphate; ceruloplasmin ^5^, copper; bilirubin -- Arylsulfatase activity ^6^, homocysteine, long-chain fatty acids ^7^, chitotriosidase activity ^8^ -- Mutation analysis in NPC1/2 gene ^8^, mutation search, and MLPA analysis GLA gene ^3^
	**Thyroid hormones**	-- TSH, free T3, free T4	
**Vitamins/trace elements**	**Vitamins**	-- Vitamin D -- Folic acid (vit. B9), cobalamin (vit. B12)	-- Thiamine (vit. B1), niacin (vit. B3), pyridoxine (vit. B6) -- Holotranscobalamin, methylmalonic acid
	**Trace elements**	-- Selenium	-- Zinc
**Pathogens**		-- Serologies for Lyme disease and lues	-- Serologies for HIV, toxoplasmosis, bartonella hanselae, TBE/FSME, EBV, hepatitis, etc.
**Immuno-logical serum screening**	**Rheumatic/immunological markers**	-- CRP, IgG/IgA/IgM, immune fixation -- CH50, C3, C4, C3d -- Rheumatoid factor ^9^	-- Interleukin 6, erythrocyte sedimentation rate, C1q complement factor, ACE ^10^, interleukin-2 receptor ^10^, neopterin ^10^, anti-streptolysin titers ^11^, CCP ^9^, HLA-B51 ^12^
	**Brain-associated systemic antibodies**	-- TRAKs ^13^, TPO/TG ^13^, and GAD65 ^14^ antibodies	-- Gliadin and transglutaminase antibodies ^15^
	**Potential antineuronal-rheumatic/other rheumatic antibodies**	-- ANA ^16^/ANCA ^17^ screening -- Anti-phospholipid (ß2 glycoprotein-I) IgG/M antibodies ^2^ -- AMA ^18^/LMA ^19^/SMA ^19^ antibodies	-- Against extractable nuclear antigens (ENA; ds-DNA, nRNP/Sm, Sm, SS-A/B, Scl-70, nucleosomes, histones, DFS70, etc.) ^16^, against PR3/MPO ^17^, against cardiolopin ^2^
	**Antineuronal serum IgG autoantibodies ^20^**	-- Against cell surface antigens: NMDA-R, AMPA-1/2-R, GABA_B_-R, LGI1, CASPR2 -- Against intracellular antigens: Yo, Hu, CV2/CRMP5, Ri, Ma1/2, SOX1, Tr, Zic4, GAD65, amphiphysin -- Against “NMO spectrum antigens”: MOG, AQP4	-- Tissue-based assays using indirect immunofluorescence (IIF) on unfixed murine brain tissue/neuropil antibodies (IIF on fixed mouse brain tissue) *--* Against other antineuronal antigens: adenylate kinase 5, DPPX, glycine-R, mGluR1/5, IgLON5, VGCC, MBP, GFAP, DRD2, etc.
**Cerebro-spinal fluid basic markers and antineuronal antibodies**	**Basic cerebrospinal fluid analyses**	-- White blood cell count, total protein, albumin quotient, IgG index, OCBs in serum/CSF, lactate	-- Glucose
	**Antineuronal IgG antibodies^20^**	*-- Against different cell surface antigens:* NMDA-R, AMPA-1/2-R, GABAB-R, LGI1, CASPR2	-- Tissue-based assays using IIF on unfixed murine brain tissue/neuropil antibodies (IIF on fixed mouse brain tissue) *-- Against intracellular antigens*: Yo, Hu, CV2/CRMP5, Ri, Ma1/2, SOX1, Tr, Zic4, GAD65, amphiphysin, etc. *-- Against other antineuronal antigens:* adenylate kinase 5, DPPX, glycine-R, mGluR1/5, IgLON5, VGCC, MBP, GFAP, DRD2, etc.
	**Infectious, neuro-degenerative and other markers**		-- MRZ reaction ^21^, antibody indices (AIs) for HSV, Borrelia burgdorferi etc.; pathogen detection of HSV, *Tropheryma whippelii*, etc.; CXCL13 ^22^ -- Tau, p-tau ^23^, ß-amyloid quotient ^23^, protein 14-3-3 ^24^, α-synuclein ^25^ -- Cytopathology and cell markers, hypocretin ^26^
**Instrument-based diagnostics**	**EEG**	-- Resting state EEG including hyperventilation period	-- Independent component analyses of the EEG -- EEG video telemetry, sleep/sleep deprivation EEG
	**Brain imaging**	-- cMRI (mostly on a 3 Tesla, rarely on a 1.5 Tesla scanner) included **T1-weighted** (on the 1.5 Tesla)/**MPRAGE** (on the 3 Tesla scanner), **FLAIR**, and **DWI** sequences	-- FDG-PET, TSPO-PET, SPECT *-- Additional cMRI sequences/methods*: T2*, T1 + contrast agent, MR-spectroscopy; resting state fMRI, DTI, etc.
**Other**	**Urine**	-- Urine status, urine drug screening, pregnancy test (for women)	-- δ-Amino-laevulinic ^27^ acid and porphobilinogen concentrations ^27^
	**Neuropsycho-logical testing**		-- Test battery for attentional performance

Diseases associated with some specific biomarkers are listed below: ^1^ neuroacanthocytosis syndromes, ^2^ antiphospholipid syndrome, ^3^ Fabry’s disease, ^4^ hyper-/hypoparathyroidism, ^5^ Wilson disease, ^6^ metachromatic leukodystrophy, ^7^ adrenoleukodystrophy, ^8^ Niemann–Pick disease type C, ^9^ rheumatoid arthritis, ^10^ sarcoidosis, ^11^ minor chorea/Pediatric Autoimmune Neuropsychiatric Disorders Associated with Streptococcal Infections (PANDAS), ^12^ Behcet’s syndrome, ^13^ autoimmune thyroiditis, ^14^ diabetes mellitus type 1/autoimmune neuropsychiatric syndromes (e.g., Stiff–Person syndrome, limbic encephalitis), ^15^ celiac disease/cerebellar degeneration, ^16^ systemic connective tissue disorders, ^17^ vasculitides, ^18^ primary biliary cirrhosis, ^19^ autoimmune hepatitis, ^20^ autoimmune encephalitis/autoimmune psychosis, ^21^ multiple sclerosis, ^22^ acute neuroborreliosis, ^23^ Alzheimer’s disease, ^24^ Creutzfeldt–Jakob disease, ^25^ synucleinopathies (Parkinson’s disease, dementia with Lewy bodies, multisystem atrophy), ^26^ narcolepsy, ^27^ acute intermittent porphyria.

**Table 2:** Sociodemographic and psychopathological findings in patients with schizophrenia spectrum disorders (SSDs). * Following the criteria of the German “Arbeitsgemeinschaft für Methodik und Dokumentation in der Psychiatrie“ (AMDP).

	Patients with SSDs (*N* = 76)
**Age in years** ± **SD (range)**	37.22 ± 14.10 (19–82 years)
**Sex**	32 males/44 females
**Highest level of education**	
Low degree	15/76 (20%)
Medium degree	21/76 (28%)
High degree	22/76 (29%)
University degree	6/76 (8%)
Unknown	12/76 (16%)
**Present professional situation**	
Unemployed	17/76 (22%)
Pension (retirement, disability)	15/76 (20%)
In training/study	17/76 (22%)
Unskilled/semiskilled work	1/76 (1%)
Qualified/first labour market	14/76 (18%)
Unknown	12/76 (16%)
Abnormal psychopathological findings *	
Attention and memory	63/76 (83%)
Formal thought	61/76 (80%)
Fear and compulsion	53/76 (70%)
Delusions	58/76 (76%)
Hallucinations	41/76 (54%)
Ego-environment boundary	32/76 (42%)
Affectivity	64/76 (84%)
Energy and psychomotor domain	64/76 (84%)
Circadian rhythm	22/76 (29%)
Suicidal tendency	14/76 (18%)
**Global Assessment of Functioning (GAF) score**	34 ± 15.54 (*N* = 65)
**Affected by disorder according to Clinical Global Impression (CGI) scores**	
Moderately affected	2/76 (3%)
Clearly affected	17/76 (22%)
Seriously affected	39/76 (51%)
Extremely seriously affected	8/76 (11%)
Unknown	10/76 (13%)
**Psychopharmacological treatment**	
Atypical neuroleptics	58 (76%)
Typical neuroleptics	4 (5%)
Antidepressants	7 (9%)
Lithium	8 (11%)
Anticonvulsants (excluding benzodiazepines)	3 (4%)
Benzodiazepines	7 (9%)
Melatonin	2 (3%)
L-thyroxine	8 (11%)
**Overall psychopharmacological treatment**	**63 (83%)**

**Table 3:** Basic laboratory analyses in patients with schizophrenia spectrum disorders (SSDs). Values with alterations in >10% are marked in bold, parameters with changes in >5% are written in bold and italics.

	Patients with SSDs (*N* = 76)
**Differential blood count**	
Leukocytes/µL (mean ± SD)	6.61 ± 1.90
Increased/decreased leukocytes (ref. 4.0–10.4 Tsd/µL)	2↑ (3%), 4↓ (5%), 70 normal (92%)
Platelets (mean ± SD)	253.57 ± 56.65
***Increased/decreased platelets (ref. 146–328 Tsd/µL)***	***6↑ (8%), 1↓ (1%), 69 normal (91%)***
Hemoglobin (mean ± SD)	14.14 ± 1.34
**Increased/decreased hemoglobin (ref. 11.6–15.5 g/dL)**	**11** **↑ (14%), 1** **↓ (%), 64 normal (84%)**
Hematocrit (mean ± SD)	40.48 ± 3.50
***Increased/decreased hematocrit (ref. 34.6–45.3 fL)***	***5↑ (7%), 7↓ (9%), 64 normal (84%)***
Neutrophil granulocytes	56.13 ± 9.34
Increased/decreased neutrophil granulocyte rate (ref. 40–75%)	1↑ (1%), 4↓ (5%), 71 normal (93%)
Lymphocytes (mean ± SD)	32.54 ± 8.95 (*N* = 75)
***Increased/decreased lymphocyte rate (ref. 19–51%)***	***3↑ (4%), 5↓ (7%), 67 normal (88%)***
**Coagulation**	
Quick (mean ± SD)	99.06 ± 15.71 (*N* = 72)
Increased/decreased quick (ref. 70–130%)	0↑ (0%), 2↓ (3%), 70 normal (97%)
INR (mean ± SD)	1.02 ± 0.22 (*N* = 75)
Increased/decreased INR (ref. 0.85–1.15)	2↑ (3%), 1↓ (1%), 72 normal (96%)
PTT (mean ± SD)	30.36 ± 5.61 (*N* = 75)
***Increased/decreased PTT (ref. 25.1–37.7 s)***	***4↑ (5%), 7↓ (9%), 64 normal (85%)***
**Electrolytes**	
Sodium (mean ± SD)	140.68 ± 2.00
Increased/decreased sodium (ref. 136–145 mmol/L)	1↑ (1%), 1↓ (1%), 74 normal (97%)
Potassium (mean ± SD)	4.36 ± 0.27
Increased/decreased potassium (ref. 3.5–5.1 mmol/L)	1↑ (1%), 0↓ (0%), 75 normal (99%)
Calcium (mean ± SD)	2.39 ± 0.16 (*N* = 75)
**Increased/decreased calcium (ref. 2.15–2.5 mmol/L)**	**9↑ (12%), 1↓ (1%), 65 normal (87%)**
Magnesium (mean ± SD)	0.85 ± 0.06 (*N* = 72)
Increased/decreased magnesium (ref. 0.66–1.07 mmol/L)	0↑ (0%), 0↓ (0%), 72 normal (100%)
**Metabolic markers**	
Creatinine (mean ± SD)	0.88 ± 0.15
Increased/decreased creatinine	60 < 1 (79%), 16 < 1.5 (21%), 0 ≥ 1.5 (0%)
α-Galactosidase (mean ± SD)	7.80 ± 3.64 (*N* = 70)
**Increased/decreased α-galactosidase (ref. 3.4–13 nmol/h/mL)**	**7↑ (10%), 8↓ (11%), 55 normal (79%)**
CK (mean ± SD)	142.70 ± 204.91 (*N* = 73)
**Increased CK (ref. < 170 U/L)**	**11↑ (15%), 62 normal (85%)**
GOT (mean ± SD)	26.97 ± 19.45
**Increased/decreased GOT (ref. 10–35 U/L)**	**10↑ (13%), 0↓ (0%), 66 normal (87%)**
GPT (mean ± SD)	32.34 ± 36.74
**Increased/decreased GPT (ref. 10–35 U/L)**	**20↑ (26%), 5↓ (7%), 51 normal (67%)**
Alkaline phosphatase (mean ± SD)	80.13 ± 32.69 (*N* = 70)
**Increased/decreased alkaline phosphatase (ref. 35–105 U/L)**	**11↑ (16%), 0↓ (0%), 59 normal (84%)**
Gamma-GT (mean ± SD)	28.83 ± 26.32 (*N* = 75)
**Increased gamma-GT (ref. < 40 U/L)**	**13↑ (17%) 62 normal (83%)**
Lipase (mean ± SD)	32.78 ± 12.57 (*N* = 68)
Increased/decreased lipase (ref. 13–60 U/L)	1↑ (1%), 1↓ (1%), 66 normal (97%)
HbA1C (mean ± SD)	5.40 ± 0.42 (*N* = 72)
Increased HbA1C (ref. 3.4–6%)	3↑ (4%) 0↓ (0%) 69 normal (96%)
Total triglycerides (mean ± SD)	145.23 ± 84.05 (*N* = 70)
**Increased total glycerides (ref. < 150 mg/dL)**	**25↑ (36%), 45 normal (64%)**
**Thyroid hormones**	
TSH (mean ± SD)	2.28 ± 1.40
Increased/decreased TSH (ref. 0.27–4.20 µU/mL)	4↑ (5%), 3↓ (4%), 69 normal (91%)
Free T3 (mean ± SD)	4.71 ± 0.71
Increased/decreased free T3 (ref. 3.1–6.8 pmol/L)	0↑ (0%), 0↓ (0%), 76 normal (100%)
Free T4 (mean ± SD)	16.51 ± 3.11
***Increased/decreased free T4 (ref. 12–22 pmol/L)***	***2↑ (3%), 5↓ (6%), 69 normal (91%)***

**Table 4:** Vitamins and trace elements in patients with schizophrenia spectrum disorders (SSDs). Values with alterations in >10% are marked in bold, parameters with changes in >5% are written in bold and italics.

	Patients with SSDs (*N* = 76)
**Vitamins**	
25-OH-Vitamin D2/D3 (mean ± SD)	22.16 ± 10.71 (*N* = 74)
**Increased/decreased vitamin D levels (ref. > 20 ng/mL)**	**34↓ (46%), 40 normal (54%)**
**Optimal vitamin D levels (ref. > 30 ng/mL)**	**62↓ (84%), 12 optimal (16%)**
Folic acid (mean ± SD)	8.80 ± 7.16 (*N* = 75)
**Increased/decreased folic acid (ref. 4.8–37.3 ng/mL)**	**0↑ (0%), 24↓ (32%), 51 normal (68%)**
Vitamin B12 (mean ± SD)	523.01 ± 303.49 (*N* = 75)
**Increased/decreased vitamin B12 (ref. 197–771 pg/mL)**	**8↑ (11%), 1↓ (1%), 66 normal (88%)**
**Trace elements**	
Selenium (mean ± SD)	64.62 ± 17.01 (*N* = 71)
**Increased/decreased selenium (ref. 75–140 µg/L)**	**0↑ (0%), 57↓ (80%), 14 normal (20%)**
**Vitamins and trace elements overall**	**69 reduced (92%), 6 normal (8%) (*N* = max. 75)**

**Table 5:** Pathogen diagnostics in patients with schizophrenia spectrum disorders (SSDs). Values with alterations in >10% are marked in bold. * Activity parameters were negative. Lues-TPPA-positive, Lues-FTA-Abs immunoglobin (Ig)M negative, Lues-FTA-Abs IgG-positive, Lues cardiolipin agglutination-negative.

	Patients with SSDs (*N* = 76)
**Serology for Lyme disease**	
IgM-ELISA screening (ref. up to 5 units)	3↑ (4%), 64 normal (89%), 5 borderline (7%) (*N* = 72)
**IgG-ELISA screening (ref. up to 16 RE)**	**10↑ (14%), 60 normal (83%), 2 borderline (3%) (*N* = 72)**
Western blot confirmation test (only performed if ELISA screening was conspicuous)	5 positive (7%), 14 negative (*N* = 19) -- IgM: negative: 8, positive: 0 -- IgG: negative: 7, positive: 5
**Serology for lues**	
Screening using CLIA (ref. < 0.9)	1 *↑ (1%), 72 normal (99%) (*N* = 73)

**Table 6:** Immunological findings in serum of patients with schizophrenia spectrum disorders (SSDs). Values with alterations in >10% are marked in bold, parameters with changes in >5% are written in bold and italics. * Abnormal findings of the immune fixation in detail: two patients with polyclonal IgA propagation, one with polyclonal IgG propagation, two with polyclonal IgM propagation, one trace evidence of a monoclonal gammopathy of the type IgG lambda.

	Patients with SSDs (*N* = 76)
**Rheumatic/immunological markers**	
**Increased C-reactive protein (ref. < 5 mg/L)**	**13↑ (17%), 62 normal (83%) (*N* = 75)**
IgG levels (mean ± SD)	10.59 ± 1.91 (*N* = 72)
IgG levels increased/decreased (ref. 7–16 g/L)	1↓ (1%), 1↑ (1%), 70 normal (97%) (*N* = 72)
IgA levels (mean ± SD)	2.09 ± 0.906
IgA levels increased/decreased (ref. 0.70–4 g/L)	1↓ (1%), 3↑ (4%), 68 normal (94%) (*N* = 72)
IgM levels (mean ± SD)	0.99 ± 0.55
***IgM increased/decreased (ref. 0.40–2.30 g/L)***	***4↓ (6%), 3↑ (4%), 65 normal (90%) (N = 72)***
Immunofixation (screening)*	6 altered (8%)*, 66 normal (92%) (N = 72)
**CH50 (ref. 65–115%)**	**1↓ (2%), 18↑ (27%), 47 normal (71%) (*N* = 66)**
**C3 (ref. 0.90–1.80 g/L)**	**11↓ (15%), 0↑ (0%), 61 normal (85%) (*N* = 72)**
C4 (ref. 0.10–0.40 g/L)	1↓ (1%), 0↑ (0%), 71 normal (99%) (*N* = 72)
Rheumatoid factor (ref. < 16 IE/mL)	73 normal (100%) (*N* = 73)
**C3d (ref. < 9 mg/L)**	**8↑ (12%), 61 normal (88%) (*N* = 69)**
**Rheumatic markers overall**	**45 positive (60%), 30 negative (40%) (*N* = max. 75)**
**Brain-associated systemic antibodies**	
TRAKs (ref. < 1.75 IU/L)	1↑ (1%), 72 normal (98%) (*N* = 73)
**Anti-TPO antibodies (ref. < 34 IU/mL)**	**9↑ (12%), 66 normal (88%) (*N* = 75)**
Anti-TG antibodies (ref. < 115 IU/mL)	3↑ (4%), 71 normal (96%) (*N* = 74)
Anti- GAD65 antibodies measured by RIA	1↑ (1%), 1 borderline (1%), 68 normal (97%) (*N* = 70)
**Anti-thyroid and diabetes antibodies overall**	**12 positive (16%), 63 negative (84%) (*N* = max. 75)**
**Potential antineuronal-rheumatic and other rheumatic serum antibodies**	
***Anti-phospholipid/ß2GP IgG antibodies (ref. < 14 GPL-U/mL)***	***5↑ (7%), 68 normal (93%) (N = 73) (range from 15 to 19 GPL-U/mL)***
Anti-phospholipid/ß2GP IgM antibodies (ref. < 10 MPL-U/mL)	3↑ (4%), 69 normal (96%) (*N* = 72) (range from 1 to 32 MPL-U/mL)
**ANA-Hep-2 (against nucleus)**	**Positive 9 (12%), negative 65 (88%) (*N* = 74)** *(In detail: trace finely spotted: 2; (+) spotted: 1 +; spotted: 1; trace homogeneous: 2; (+)-+ homogeneous: 1; (+) homogeneous: 1; + homogeneous-fine-spotted: 1)*
ANA-Hep-2 (nucleoli)	Positive 0 (0%), negative 74 (100%) (*N* = 74)
ANA-Hep-2 (chromosomes)	Positive 4 (5%), negative 70 (95%) (*N* = 74) (In detail: trace: 1, (+): 1, + stripy: 1, (+)-+ homogeneously: 1)
ANA-Hep-2 (cytoplasm)	Positive 2 (3%), negative 72 (97%) (*N* = 74) *(In detail: Trace reticular: 2)*
**ANA overall positive/borderline findings**	**Positive 11 (15%), negative 63 (85%) (*N* = max. 74)**
ENA screening (only performed if ANAs were clearly positive)	Positive 3 (33%), negative 6 (66%) (*N* = 9) *Positive cases in detail: anti-snRNP/Sm +++ in one patient, anti-DFS70 +++ in two patients; anti-centromere (CENP B) (+) in one patient, anti-AMA-M2 (IgG) (+) in one patient*
Anti-dsDNA ELISA (ref. < 40 U/mL; only performed if ANAs were clearly positive and suspicious clinical findings were present)	12.33 ± 7.02 (*N* = 3) 0↑ (0%), 3 normal (100%)
ANCAs (IgG, ref. 1:10)	2 positive (3%), 71 negative (97%) (*N* = 73) *Positive cases: n = 1 anti-MPO negative (2 U/mL; ref. <5 U/mL), anti-PR3 negative (1 U/mL; ref. <10 U/mL; in n = 1 not performed*
AMA/LKM (kidney, ref. 1:50)	0 Positive (0%), 60 negative (100%) (*N* = 60)
SMA (kidney, ref. 1:50)	4 borderline positive (7%), 56 negative (93%) (*N* = 60) *(In detail: n = 2 trace, n = 2 (+))*
**Rheumatic antibodies overall**	**23 positive (31%), 51 negative (69%) (*N* = max. 74)**
**Established antineuronal serum antibodies**	
Antibodies against intracellular onconeural antigens (*Hu, Yo, Ri, cv2(CRMP5), Ma1/-Ma2, SOX, Tr(DNER), Zic4, amphiphysin, GAD65*)	Clear positive: in 1 case (Yo; 2%) Weak, non-specific bands: in 5 cases (Yo 3x, SOX 2x, Zic4 1x; 8%) Negative: 54 (90%) (*N* = 60)
Antibodies against neuronal cell surface antigens *(LGI1, CASPR2, GABA-B, NMDA-R, AMPA 1/2*)	0 positive (0%), 56 negative (100%) (*N* = 56)
Anti-AQGP4 antibodies	0 positive (0%), 52 negative (100%) (*N* = 52)
Anti-MOG antibodies	1 positive (2%), 49 negative (98%) (*N* = 50)

**Table 7:** Cerebrospinal fluid findings in patients with schizophrenia spectrum disorders (SSDs). Values with alterations in >10% are marked in bold, parameters with changes in >5% are written in bold and italics.

	Patients with SSDs (*N* = 48)
**CSF basic analyses**	
WBC count (mean ± SD)	1.38 ± 0.67
Increased WBC count (ref. < 5/µL)	0↑ (0%), 48 normal (100%)
Protein (mean ± SD)	553.18 ± 401.82 (*N* = 45)
**Increased protein (ref. < 450 mg/L)**	**25↑ (56%), 20 normal (44%)**
Albumin quotient (mean ± SD)	6.14 ± 5.39
**Increased albumin quotient (references: <40 years: < 6.5 × 10^−3^; 40–60 years: < 8 × 10^−3^; >60 years: < 9.3 × 10^−3^)**	**10↑ (21%), 38 normal (79%)**
IgG index (mean ± SD)	0.52 ± 0.09
Number of patients with increased IgG indices (ref. < 0.7 mg/L)	2↑ (4%), 46 normal (96%)
Oligoclonal bands in CSF	47 negative (98%), 1 positive (2%)
Oligoclonal bands in serum	48 negative (100%), 0 positive (0%)
Lactate (mean ± SD)	1.60 ± 0.29 (*N* = 40)
**Increased lactate (ref. 16–50 years: 1.5–2.1 mmol/L; >51 years 1.7–2.6 mmol/L)**	**18↓ (45%), 0↑ (0%), 22 normal (55%)**
**CSF overall alterations** (without decreased lactate levels)	**26/48 (54%)**
**Established antineuronal antibodies**	
Antibodies against established cell surface antigens *(LGI1, CASPR2, GABA B, NMDA, AMPA 1* and *AMPA 2, DPPX)*	47 negative (100%), 0 positive (0%) (*N* = 47)

**Table 8:** Electroencephalography (EEG) and cerebral magnetic resonance imaging (cMRI) alterations in patients with schizophrenia spectrum disorders (SSDs). * Two patients had white matter lesions compatible with chronic inflammatory changes. One patient had multiple sclerosis-typical MRI lesions. This patient suffered from an isolated psychotic episode. She was diagnosed with multiple sclerosis in the past; however, the patient was not given prophylaxis against relapse. ** Two patients were only suspected of having a pineal cyst. Abbreviation: IRDA/IRTA, intermittent generalized rhythmic delta/theta activity; HV, hyperventilation.

	Patients with SSDs
**EEG (*N* = 76)-Visual assessment**	
Continuous generalized slow activity	0/76 (0%)
Continuous regional slow activity	1/76 (1%)
Intermittent generalized slow activity	16/76 (21%)
Intermittent regional slow activity	6/76 (8%)
Epileptic pattern	0/76 (0%)
**EEG overall alterations**	**22/76 (29%)**
**EEG (*N* = 76)—Automatic IRDA/IRTA quantification (mean values)**	
IRDA/IRTA rate before hyperventilation	1.18 ± 1.79 (*N* = 76)
IRDA/IRTA rate after hyperventilation	1.64 ± 2.23 (*N* = 66, hyperventilation was not performed in all patients)
IRDA/IRTA difference (post-hyperventilation–pre-hyperventilation)	0.46 ± 1.59 (*N* = 66)
**IRDA/IRTA rates overall**	**1.27 ± 1.70 (*N* = 76)**
**cMRI (*N* = 74)—Visual assessment**	
(Non-specific) white matter lesions	28/74 (38%)
Chronic inflammatory lesions *	3/74 (4%)
Atrophy	1/74 (1%)
Pineal cyst **	12/74 (16%)
Others	19/74 (26%)
**cMRI overall alterations**	**45/74 (61%)**
